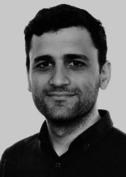# Interfacing Living and Synthetic Cells as an Emerging Frontier in Synthetic Biology

**DOI:** 10.1002/ange.202006941

**Published:** 2020-10-13

**Authors:** Yuval Elani

**Affiliations:** ^1^ Department of Chemical Engineering Imperial College London Exhibition Road London UK

**Keywords:** artificial cells, biotechnology, cellular bionics, molecular bioengineering, synthetic biology

## Abstract

The construction of artificial cells from inanimate molecular building blocks is one of the grand challenges of our time. In addition to being used as simplified cell models to decipher the rules of life, artificial cells have the potential to be designed as micromachines deployed in a host of clinical and industrial applications. The attractions of engineering artificial cells from scratch, as opposed to re‐engineering living biological cells, are varied. However, it is clear that artificial cells cannot currently match the power and behavioural sophistication of their biological counterparts. Given this, many in the synthetic biology community have started to ask: is it possible to interface biological and artificial cells together to create hybrid living/synthetic systems that leverage the advantages of both? This article will discuss the motivation behind this cellular bionics approach, in which the boundaries between living and non‐living matter are blurred by bridging top‐down and bottom‐up synthetic biology. It details the state of play of this nascent field and introduces three generalised hybridisation modes that have emerged.

## Graphical abstract

This Minireview outlines the state of play in the research area of cellular bionics, where biological and synthetic cells are hybridised, thus bridging the living/non‐living divide.

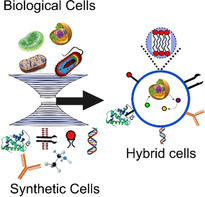

## 1 Introduction

The field of synthetic biology concerns itself with the design of biological systems not found in the natural world.^[1]^ This allows the power of biological systems, honed over evolutionary history, to be tapped into and ultimately engineered for functional applications. This approach also leads to new insights into the “rules of life”: understanding biology by building a new biology.^[2]^ Synthetic biology has been heralded as one of the critical emerging technologies of the 21st century, offering solutions to diverse real‐world problems. From combatting climate change through the production of biofuels,^[3]^ to removing pollutants via bioremediation, to using engineered cells as therapeutics^[4]^ and in regenerative medicine.[^1^, ^5^]

**Figure 1:**
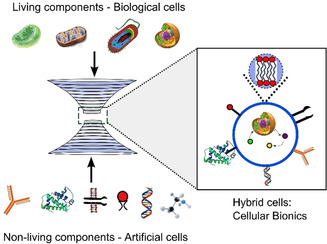
Cellular bionics. Hybrid cellular bionic systems can be constructed by fusing living and non‐living modules together. Living modules can be cells (prokaryotic or eukaryotic) or organelles (e.g. mitochondria and chloroplasts). Non‐living modules can consist of artificial cell‐like compartments that are composed of biological and synthetic molecular components (e.g. enzymes, membrane proteins, DNA, and nanoparticles).

Synthetic biology has traditionally been divided into two distinct approaches. The first is “top‐down”, where cells are modified using molecular biology and metabolic/genetic engineering techniques. The alternative approach is concerned with constructing cell‐like structures known as artificial cells (also known as protocells or synthetic cells) from scratch out of non‐living building blocks. This endeavour is sometimes referred to as “bottom‐up” synthetic biology.^[6]^ The ultimate aim here is to engineer new cell‐like entities from inanimate matter. This perspective focuses on the space in between the two approaches: the construction of composite structures in which biological and artificial cells are intermingled to create hybrid systems composed of living and synthetic components (Figure 1).

As a field, top‐down synthetic biology is well developed and has already produced several breakthroughs including the biosynthesis of drug precursors,^[7]^ the development of organisms for biofuel production,^[3]^ engineered cell therapies,^[8]^ and the creation of new responsive and multifunctional materials.^[9]^ By contrast, the discipline of artificial cells is less mature in terms of demonstrated applications. Concepts and methods in this area were first developed in the 1990s by pioneers such as Luisi, Yomo, and others,^[10]^ who laid the foundations for the remarkable growth of this research area over the past decade. There are now several dedicated large‐scale international centres and initiatives devoted to building artificial cells from the bottom up.^[11]^

The most dominant form of artificial cells involve cell‐sized capsules, such as liposomes, polymersomes, coacervates, proteinosomes and hydrogel particles, which act as the chassis.[^6a^, ^12^] These compartments can be functionalised with biomolecular components, including transmembrane channels,^[13]^ enzymes,^[14]^ cytoskeletal elements,^[15]^ gene circuits,^[16]^ and transcription/translation machinery.^[17]^ In doing so, cellular characteristics can be mimicked.[^6^, ^18^] These include cellular processes and behaviours (e.g. signalling cascades,^[19]^ communication,^[16]^ motility,^[20]^ energy generation,^[21]^ replication,^[22]^ and computation)^[23]^ as well as architectural motifs (e.g. membranes, organelles, and tissues).^[24]^

The precise attributes that a construct must have to be considered an artificial cell is still up for debate, and definitions vary between research groups. Some consider any collection of functional biologically‐relevant molecules encased in cell‐sized capsules to be artificial cells. Others emphasise the need to mimic cellular behaviours that are considered the hallmarks of life. Other bones of contention are whether incorporation of genomic componentry are prerequisites, whether artificial cells must be composed of biologically derived building blocks, or if morphological resemblance is enough. Perhaps the strictest definitions are those which only class fully autonomous, autopoietic, self‐sustaining, replicating and evolving biochemical microsystems as artificial cells; this is seen as one end goal of the field, and is yet to be achieved. For this reason, artificial cells cannot currently be considered “alive” which is one of their attractions, as will be elaborated on later.

Beyond pure scientific curiosity, three classes of motivation drive artificial cell research. The first is related to origin‐of‐life research, where building protocellular systems helps to shed light on how life arose in the early earth;^[25]^ this was an early driver for the research in this space. The second is the use of artificial cells as models with which to study biology in a simplified and highly controlled environment.^[26]^ The third lies in their potential applications as soft, responsive micromachines, specifically engineered to perform useful biotechnological functions. Rapid developments in this area have meant that real‐world applications of artificial cells—as therapeutic agents, sensors, self‐healing materials, and biochemical microreactors—are on the horizon.

Top‐down and bottom‐up synthetic biology have largely evolved in parallel to each other, and they still exist as distinct sub‐fields with little by way of meaningful overlap. However, we are reaching a point where links between the two approaches can be made through the construction of hybrid cells composed of both living and synthetic components. This embryonic research space has emerged in part due to improved technological capabilities: platforms to form (i) adequately complex artificial cells (ii) robust enough engineered living cells, and (iii) tools to hybridise the two, have only recently come into their own. The merits of this hybridisation strategy, sometimes referred to as cellular bionics, are derived from combining the advantages associated with both approaches, which will now be detailed.

## 2 Top‐down vs. Bottom‐up: A Comparison

There are several advantages associated with engineering artificial cells from scratch rather than re‐engineering living cells. First, the limitation of cellular burden—the tug of war associated with distributing energy and resources between natural and engineered cell functions—is not a feature of synthetic cells.^[27]^ They do not have to devote resources to auxiliary processes associated with performing tasks that cells have evolved to undertake, and indeed, to staying alive. Engineered processes can thus potentially be more efficient if cells are fully synthetic. Second, the fact that synthetic cells are not living means that one can engineer them to produce otherwise toxic compounds.^[28]^ Moreover, as biocompatibility issues are no longer critical with non‐living cells, one can incorporate wholly non‐biological building blocks, allowing biological capabilities to be surpassed. These non‐biological additions could include electronic components, functional nanoparticles, and novel molecular machines (e.g. DNA origami or nano‐electrical elements). Third, the use of synthetic cells reduces biosafety, regulatory, and public perception hurdles.^[29]^ They do not replicate, are not alive, are not autonomous, and are not functional for long periods without active human intervention—at least for the foreseeable future. In this respect, they have more in common with traditional nanotechnologies and microrobots than with living organisms. For these reasons, they are not considered GMOs by either regulators or the public psyche. Finally, and perhaps most importantly, synthetic cells have a vastly reduced molecular complexity compared to their biological counterparts, which makes them more programmable and predictable.^[30]^ They are often composed of tens of distinct molecular species, compared to tens of thousands of species present in living cells. In a scenario where each biomolecule interacts with many others, this yields an interaction network with exponentially increasing complexity for every new component added. Moreover, living cells may modify their internal biochemistries to resist changes imposed on them, often in an unpredictable manner. These factors make it difficult to effectively predict how living cells will respond when re‐engineered to have new functions. Additionally, one knows (and can precisely control) the full molecular composition of synthetic cells—since they are constructed from scratch—which is not the case with living cells.

However, despite their promise, the capabilities of artificial cells are inherently limited compared to living biological ones. Biological cells have dynamic metabolic, biosynthetic, and regulatory pathways. Their molecular complexity means they are capable of energy conversion and of driving themselves out of equilibrium. They can self‐repair, interact with one another to yield collective behaviours, self‐replicate and be cultured at scale. Exploiting these features has underpinned many biotechnological advances and is predicted to be a major driver behind the “fourth industrial revolution”.^[31]^ It is clear that biological cells have a behavioural sophistication that artificial cells cannot currently match. Moreover, when it comes to producing industrially relevant quantities of products in an economically acceptable manner, it is unlikely that the performance of “living” cells will be surpassed by synthetic ones.

It is important to note that the boundary between biological and synthetic cells is not always clear‐cut, with the distinction between organism and machine being a central topic in theoretical discussion in synthetic biology.^[32]^ The border between the two is being approached from several directions, including artificial cells with increasing life‐like characteristics, minimal cells based on synthetic genomics, and genetically programmed bacterial/eukaryotic machines produced by bioengineering.^[33]^

## 3 Hybrid Cells

The simultaneous proliferation of microfluidics, cell‐free protein expression, gene circuit design, and membrane engineering technologies has meant that it is now possible to fuse living and synthetic cells together to form hybrid entities. This in turn allows the advantages associated with each to be combined and the disadvantages to be tempered. One can envisage cells and organelles being used as functional modules that are coupled to artificial cells, allowing them to harness the full power of biology. Directly using living cells as modules in an artificial cell context bypasses the limitations of making new modules from scratch. Instead, cellular components that have been sculpted through evolution can be hijacked, enabling a step‐change in artificial cell sophistication and capabilities.

**Figure 2:**
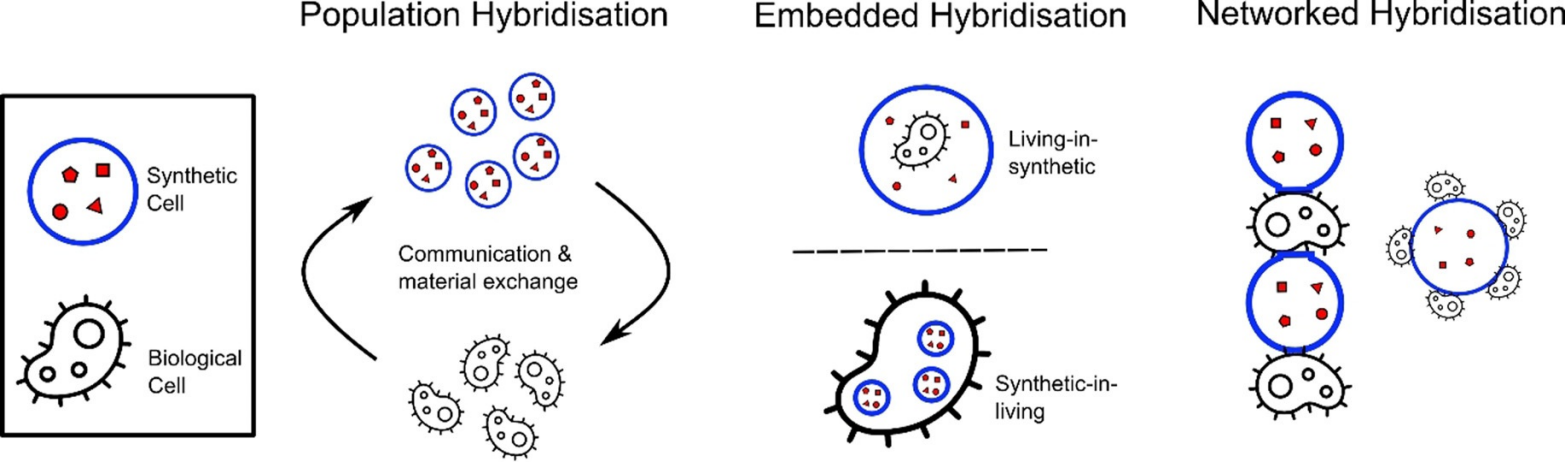
Schematic of the main cellular bionic hybridisation modes in which living and synthetic cells are chemically or physically interfaced with each other.

Although this area of research is still in its infancy, several recent studies have emerged which have allowed some generalisation to be made regarding the different modes with which living and synthetic cells can be coupled. Overall, three different hybridisation routes are possible, depicted in Figure 2.

- Population hybridisation, where discrete biological and artificial cells communicate with one another across space, exchanging information and materials.
- Embedded hybridisation, where living cells are embedded inside synthetic ones or vice versa, with the encapsulated cells performing organelle‐like functions within their host.
- Networked hybridisation, where artificial and biological cells exist as distinct entities that are physically linked to one another in a network or in a tissue‐like arrangement.

Through these three hybridisation routes, it is possible to traverse length‐scales: from the molecular to the organelle, cellular, and multi‐cellular bulk material level. Now follows a review of some landmark examples which fall within these classes.

### 3.1 Population Hybridisation

Fairly diverse literature already exists on communication between various synthetic cells of different classes, including ones based on lipid vesicles,[^16^, ^34^] proteinosomes^[23]^ and polymersomes.^[34]^ There have also been examples of engineered communication between two different species of artificial cells, including between vesicles and proteinosomes.^[12a]^ Predator/prey^[35]^ and response/retaliation^[36]^ relationships between coacervates and proteinosomes have also been demonstrated.

**Figure 3:**
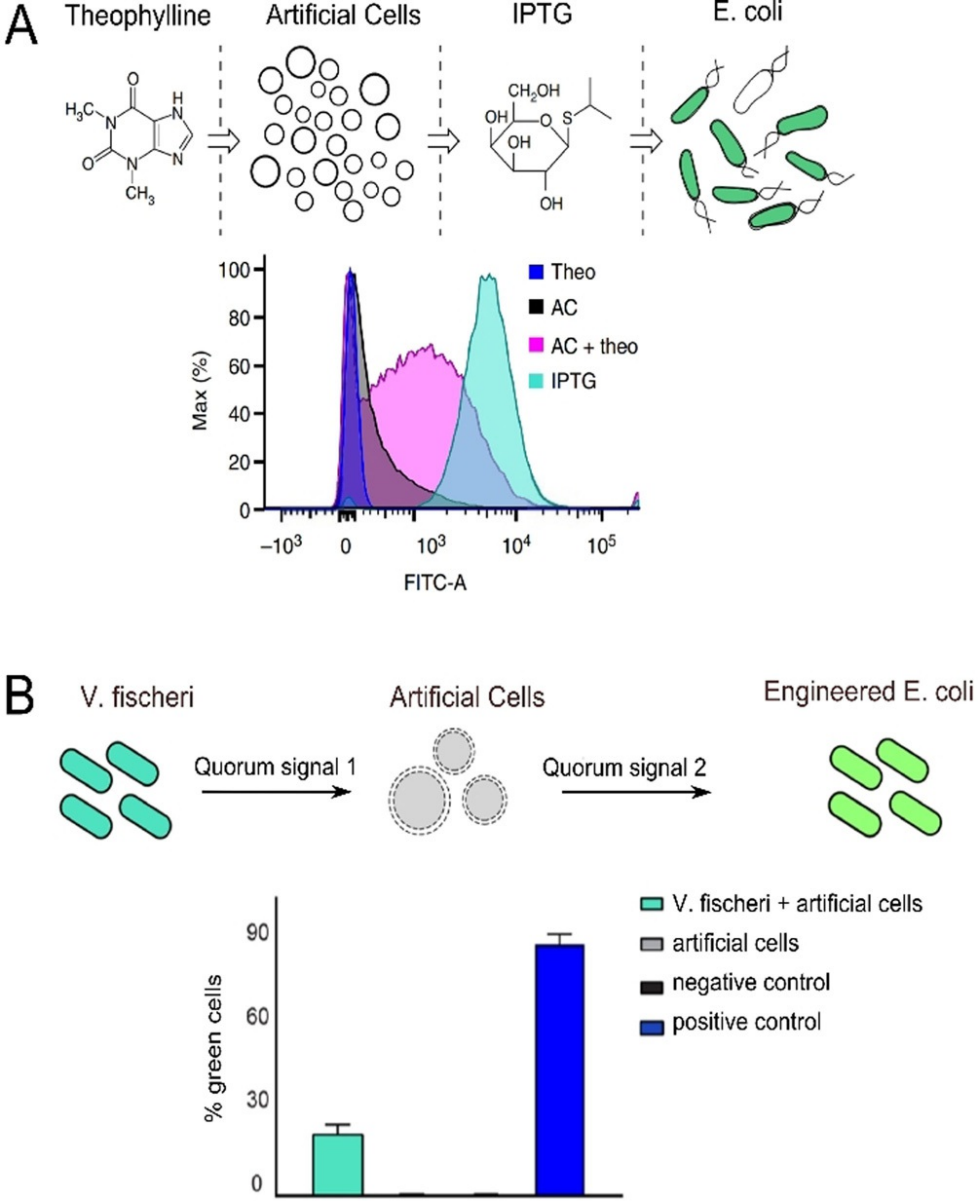
Population hybridisation. A) Schematic of artificial cells translating a chemical signal (theophylline) into a signal (IPTG) which *E. coli* can sense and respond to through GFP expression. FACS results showing a shift in fluorescence of the *E. coli* population in the presence of artificial cells plus theophylline are shown below. Image modified with permission from Ref. [39]. B) Schematic of an artificial cell mediating communication between *V. fischeri* and engineered *E. coli* through the sensing and releasing of different quorum‐sensing molecules. FACS results showing successful communication through GFP expression in *E. coli* are shown below. Image modified with permission from Ref. [40].

There have been efforts to build on these examples and engineer communication between discrete populations of living and synthetic cells (Figure 3). Some of these have required the cells to be embedded in an agar gel matrix to protect the artificial cells from the destabilising effects of surrounding bacteria.^[37]^ One early example involved the development of a synthetic cell containing a proto‐metabolism capable of synthesising a molecule that elicited a cell‐signalling response in bacteria.^[38]^ The proto‐metabolism consisted of an autocatalytic sugar‐synthesising formose reaction, the product of which was secreted from the synthetic cell and then engaged the natural quorum‐sensing mechanism of *Vibrio harveyi*, yielding a luminescent output.

This concept was taken to a new level by Lentini et al., who, instead of using enzymatic reactions, incorporated genetic elements and cell‐free expression systems in artificial cells to establish the communication pathway (Figure 3 A).^[39]^ Their vesicle‐based cells contained a DNA programme that coded for a riboswitch that activated translation in response to the presence of a small molecule, theophylline. This then initiated the synthesis of the pore‐forming protein α‐hemolysin (αHL), which led to the release of the inducer molecule IPTG, which in turn initiated GFP production in a population of *E. coli*. Theophylline is a molecule that *E. coli* would not normally respond to. The synthetic cells in effect were able to “translate” it into a chemical signal that can be processed by bacteria, thus effectively expanding the sensory range of the *E. coli*. All this was done without significantly altering the genetic content of the bacteria.

The same group,^[40]^ as well as other researchers,^[41]^ expanded on this work by engineering synthetic cells that could both send *and* receive chemical messages to/from bacteria, completing the communication loop. This was done by reconstituting cellular quorum‐sensing pathways in synthetic cells, thus achieving two‐way communication.^[40]^ This principle was also used to engineer communication among three species of cells, with the artificial cells mediating communication between two different bacterial species (Figure 3 B). Interestingly, the authors used the ability of bacteria to recognise the synthetic cells as one of their own as a yardstick to determine how “life‐like” the artificial cells are, in a similar manner to the Turing test for artificial intelligence. The authors proposed that the degree of response from the bacteria, a process that can be quantified using analysis of RNA from transcription, can be used as a proxy for how “life‐like” the artificial cells are. This nicely demonstrates some of the philosophical implications that can arise from hybridising living and synthetic cells.

Others have deployed communication between living and synthetic cell populations to demonstrate functional applications through feasibility studies. For example, Krinsky et al. have shown potential therapeutic uses for this approach through the construction of artificial cells that synthesised anti‐cancer proteins that inhibited tumour growth both in vitro and in vivo.^[42]^ Successful attempts at engineering a feedback sense/response system between artificial and living cells have also led to the synthesis of antimicrobial peptides that killed surrounding bacteria through lysis.^[41]^ Finally, Amidi et al. have explored the use of artificial cells as genetically programmable vaccine microreactors through cell‐free protein expression of antigens.^[43]^ When deployed in mice, these were shown to elicit an immune response.

### 3.2 Embedded Hybridisation

**Figure 4:**
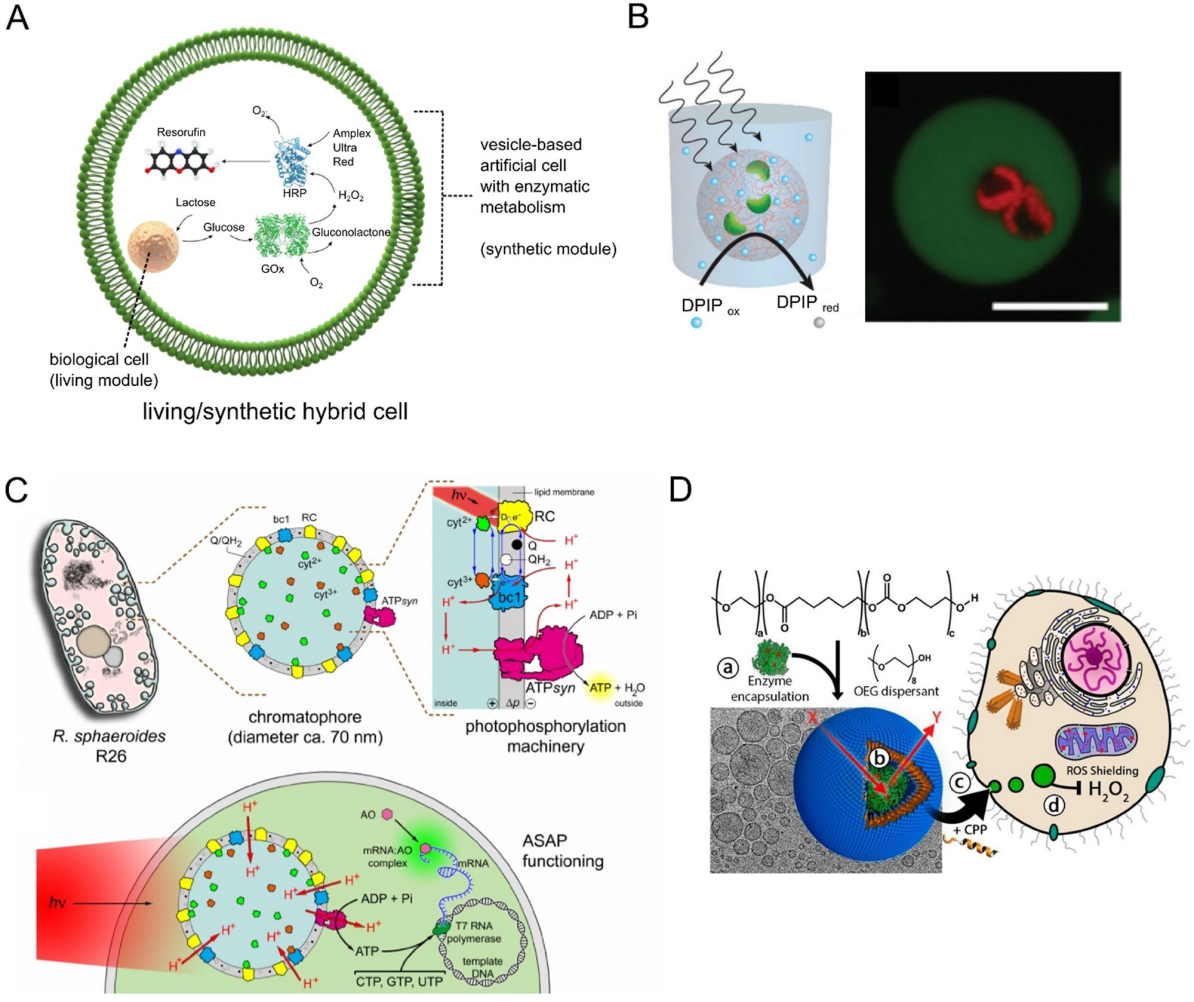
Embedded hybridisation. A–C) Examples of living cells encapsulated in synthetic ones. A) Engineered eukaryotic cells encapsulated in an artificial cell containing a synthetic metabolism. Coupling the living and synthetic cells in this way resulted in an enzymatic cascade leading to the production of a fluorescent molecule (resorufin) within the hybrid bioreactor. Figure modified with permission from Ref. [47]. B) Microscopy images showing chloroplasts (red) encapsulated in coacervates (green). Chloroplasts retained their light‐induced electron transport capabilities, as demonstrated by the reduction of a Hill reagent (DPIP), depicted in the schematic. Figure modified with permission from Ref. [50]. C) Schematic of a chromatophore organelle extracted from a photosynthesising organism and inserted in a synthetic cell. Upon light irradiation, this led to the production of ATP, which powered the translation apparatus to produce mRNA. Figure modified with permission from Ref. [51]. Copyright 2020, the authors. D) An example of a synthetic cell encapsulated in a living one. The synthetic cells performed an organelle‐like function by degrading H_2_O_2_, thus shielding the cell from the detrimental effects of this molecule. Image modified with permission from Ref. [54].

The embedded hybridisation mode involves the creation of hybrid cells through physical encapsulation (Figure 4). This could either involve encapsulating living cells within synthetic ones, or vice versa. An analogy can be drawn with the origin of eukaryotic organelles, where previously free‐living organisms were taken up by a host to yield eukaryotic cells through a mutually beneficial symbiotic relationship between the two. The resulting organelles exist in a distinct physicochemical environment, allowing them to be specialised to perform specific tasks. Embedded organelle‐like compartments have been used in a purely artificial cell context, for example for spatially segregated transcription and translation,^[44]^ for stimuli‐responsive enzymatic reaction in vesicle‐based cells,^[45]^ and for engineering synthetic signalling cascades between compartments.^[46]^ There is now an emerging trend to expand this concept for the creation of hybrid living/synthetic systems.

#### 3.2.1 Biological Cells Encapsulated in Synthetic Cells: “Living‐in‐Synthetic”

There have been several demonstrations of living cells encapsulated in synthetic ones for the creation of hybrids. For example, droplet microfluidics was used to encapsulate cells in giant lipid vesicles, with the encapsulated cells engineered to have an organelle‐like function.^[47]^ Specifically, colon carcinoma cells were modified to express an enzyme which performed one step of a multi‐step enzymatic cascade (Figure 4 A). The enzymatic product was then further processed by a synthetic metabolism co‐encapsulated in the vesicle. In this way, the encapsulated cell acted as a bioreactor module within the synthetic cells. This approach was expanded further through the encapsulation of genetically engineered *E. coli* designed to sense lactate within vesicles.^[48]^ The bacteria thus acted as a biosensor module, allowing the detection of lactate by the hybrid vesicle construct. In these examples, not only did the biological cell confer functionality to the synthetic cell, but the synthetic cell also shielded the encapsulated cell from a toxic exterior, demonstrating a mutually beneficial relationship. Other researchers have encapsulated mitochondria in vesicles^[49]^ and viable chloroplasts in coacervate‐based synthetic cells (Figure 4 B),^[50]^ leading to the exciting possibility of these organelles being used as biobattery modules to power encapsulated biochemical processes. Finally, in a recent breakthrough paper, Altamura et al. extracted chromatophores from *Rhodobacter sphaeroides* and used them as photosynthesising organelles within artificial cells (Figure 4 C). Under illumination, these converted ADP to ATP, which in turn sustained the transcription of DNA to mRNA, paving the way for continual regeneration of energy‐carrying molecules using an external energy source.^[51]^ Similarly, there have been recent efforts to appropriate thylakoid membranes and to couple these to a synthetic enzymatic cycle that fixes carbon dioxide within water‐in‐oil droplets, thus achieving a photosynthetic anabolic reaction in a cell‐like construct.^[52]^

#### 3.2.2 Synthetic Cells Encapsulated in Biological Cells: “Synthetic‐in‐Living”

The architectural motif above can also be reversed, with synthetic organelles being introduced into living cells, as a form of cellular implant. To date, synthetic organelles have relied on enzymatic processes and do not operate with DNA programmes coupled with cell‐free protein expression. One impressive example of synthetic‐in‐living hybrids involved the creation of polymersome‐based synthetic organelles that housed enzymes capable of producing a fluorescent molecule from a non‐fluorescent precursor.^[53]^ These organelles were designed to be stimuli‐responsive: molecular flow through the engineered protein pore OmpF (and hence activation of the organelle) was triggered when the structure experienced a change in redox potential as it entered the intracellular microenvironment. Impressively, it was shown that these organelles could respond to intracellular glutathione levels and were functional in vivo in zebrafish embryos.

Similarly, van Oppen et al. used a polymersome‐based system that was functionalised with cell‐penetrating peptides on its outer surface, which facilitated uptake by human embryonic kidney cells as well as human primary fibroblasts.^[54]^ The organelles housed the enzyme catalase in the interior, which allowed them to degrade externally added reactive oxidative species, shielding the cell from their negative effects (Figure 4 D). A similar approach, which relied on polymersomes functionalised with transmembrane channels and two coupled enzymes, was used to mimic peroxisomes. These organelles were able to detoxify both superoxide radicals and H_2_O_2_. These examples are further demonstrations of the potential of organelle therapy, given that reactive oxidative species in general, and reduced catalase activity in particular, are thought to play a role in a host of medical conditions including Alzheimer's, Parkinson's, Huntington's, acatalasemia, metabolic diseases, and cancer.^[55]^

Other researchers have developed multi‐layered synthetic organelles composed of both liposomes and polymersomes that housed distinct enzymes in different layers.^[56]^ These were able to perform multi‐step enzymatic cascades with glucose as a feedstock. The synthetic organelles could be internalised by macrophages, where they preserved their activity, utilising an intracellular source of glucose to initiate a controlled foreign cascade reaction in the host cell. There has also been an example of synthetic organelles that were designed to *reduce* cell viability through the production of reactive oxidative species using the enzyme glucose oxidase and a glucose feedstock.^[57]^

Finally, in a recent study, a suite of synthetic vesicle‐based organelles with diverse functionalities formed using droplet microfluidics were incorporated in living cells.^[58]^ One organelle type mimicked peroxysomes through the incorporation of enzymatic modules. A second organelle type was engineered to act as an intracellular light‐responsive signalling module that could release calcium stores, with the potential to act as an artificial regulatory element. A third type of organelle was designed to impart the host with a magnetotactic sense, allowing cells to reorient themselves and move in response to a magnetic field. The latter example is a powerful demonstration of the incorporation of entirely new, non‐intrinsic functionalities not otherwise found in host cells.

### 3.3 Networked Hybridisation

The networked hybridisation mode involves discrete artificial and biological cells physically interlinked with one another in a distinct spatial arrangement. To date, there have been only a handful of examples of this, outlined below. This is in contrast to interlinked networks of purely synthetic cells, of which there are more examples, including the creation of self‐folding tissue‐like networks composed of thousands of bilayer‐linked compartments,^[59]^ light‐activated gene‐expression in individual cells of a synthetic tissue,^[60]^ networked synthetic cell compartments for controlled prodrug activation and release,^[61]^ and thermoresponsive proteinosome clusters capable of cyclical expansion and contraction.^[30c]^

One of the few examples of hybrid living/synthetic networked structures involved artificial and biological cells that communicated with each other in a manner dependent on their precise geometrical connectivity.^[62]^ In this work, the networked compartments were based on water‐in‐oil droplets that were connected in a linear chain. Droplets in the network either contained living *E. coli* or a cell‐free expression system and a DNA programme. Both the synthetic and biological cells were engineered to possess genomes that could produce inducer molecules or respond to them through GFP expression, with the sensing mechanism regulated through the lux operon. The authors were able to obtain position‐dependent gene expression using a morphogen gradient, thus creating a form of artificial cell differentiation that was determined by their location in relation to their neighbours.

Another example involved the use of acoustic standing wave patterning to link up vesicle‐based synthetic cells and bacteria.^[63]^ The technology enabled the geometries, lattice dimensions, and trap occupancy of structures to be controlled. Using this setup, the authors could form networks of synthetic cells that produced H_2_O_2_ through an enzymatic cascade. This reaction product then diffused through embedded protein pores to adjacent *E. coli* cells, leading to cell death. This approach was extended to engineer communication between 1D and 2D networks of synthetic cells and red blood cells.^[64]^ Similar systems involving positively charged proteinosomes, which show a programmed temperature‐ and salt‐dependent interaction with living *E. coli*, have been developed.^[65]^ These were shown to be able to capture and release microbes from the colloid surface in a stimuli‐responsive manner. Finally, there have been efforts at generating extended tissue‐like hybrid materials by using 3D printing of synthetic tissues composed of lipid‐membrane‐coated synthetic cell chassis. These constructs contained embedded mammalian cells, an approach that allows high‐solution patterning of cells within a printed material and has potential applications in regenerative medicine.^[66]^

## 4 Conclusions

The field of living/synthetic hybrid cellular systems is still very much in a nascent stage, with most studies relying on the development of underpinning technologies and proof‐of‐concept experiments. The field is probably 15 years or so from reaching the maturity needed for true applications to be realised. What is already clear, however, is that combining synthetic cells with living cells could be strategically important to the fields of cellular and molecular bioengineering. It will drive innovation and widen synthetic biology's application base, allowing cells to be coupled with artificial microsystems that include electronic, mechanical, and chemical components.

Potential biotechnological and biomedical applications are wide and diverse: from cell therapies shielded by an artificial membrane delivery chassis, to chemical microsystems powered by photosynthesis, to self‐healing materials that use biosynthetic pathways to regenerate building blocks, to hybrid chemo/bioreactors. However, before such applications can be realised, the engineering routes to interlink biological and synthetic cells need to be devised, which is the current focus of activities in this area. Moreover, for the field to realise its potential, the creation of hybrid living/synthetic systems need be not only possible, but also affordable, scalable, and adaptable for different applications. There are also some structural issues which have stymied progress in this area. Research in top‐down and bottom‐up synthetic biology typically belong to different scientific domains, and are housed in different university departments: the former tend to lie in life sciences and bioengineering (molecular biology, metabolic engineering, cell biology) and the latter in the physical sciences (soft matter, biophysics, microfluidics, chemical biology, chemical engineering).

At this point, it should be noted that there are limitations to this hybridisation approach. Most importantly, while combining the different advantages associated with living and synthetic systems, one would also be in danger of accumulating the disadvantages. For this reason, hybrid cellular systems will be more suited for particular applications than others. As the field advances, mitigation strategies to minimise the downsides are expected to be developed.

There is a timeliness to this research challenge, given the unique opportunities derived from the proliferation of physical science innovations related to this area. These include microfluidic devices for the high‐throughput manufacture of cell‐sized vesicles and other compartmentalised structures[^24^, ^67^] as well as new methods for the efficient encapsulation of biological macromolecules in membrane capsules.^[49]^ New optical trapping technologies have allowed the manipulation of cell‐sized objects and assembly of user‐defined biomimetic architectures.^[68]^ Laser‐based approaches for the spatial patterning of tissue‐like materials with fine spatio‐temporal resolution have also been elegantly demonstrated,^[69]^ and there are growing synergies between electronic and living cellular systems.^[70]^ Moreover, rapid developments in DNA nanotechnology^[71]^ and protein engineering^[72]^ will further expand the repertoire of building blocks which can be used to interface synthetic and living cells.

From the bio‐science sector, the rise of commercial cell‐free expression kits, cheap and portable DNA sequencing,^[73]^ online repositories of DNA “biobrick” genetic components, and gene synthesis services will no doubt continue to drive the development of hybrid cellular systems. Moreover, many of the processes involved have been effectively “deskilled” with the evolution of biohackspaces and the growing ubiquity of 3D printers and bio‐printers.^[74]^ There are also an ever‐expanding array of commercial ready‐to‐use biochemical systems for enzymatic assays, cell‐free protein expression, and synthetic biology education.^[75]^ All this means that technology development and applications are no longer restricted to specialised microfluidics groups, and biochemical functionalisation of synthetic constructs is not confined to life‐science labs, which aids this inherently multidisciplinary endeavour.

In conclusion, given the pace of change in this area, it is expected that hybrid living/synthetic cellular bionic systems will rapidly increase in number and complexity over the coming years. There will no doubt be unexpected hurdles along the way, but there are already indications that this will emerge as a distinctive and disruptive research area that bridges the life, physical, and engineering sciences. Not only will it lead to diverse applications, but it also has fascinating philosophical implications: blurring the boundary between living and non‐living matter will change our perception of what it means for something to be alive.

## Conflict of interest

The author declares no conflict of interest.

## Biographical Information

*Yuval Elani is a UKRI Future Leaders Fellow and Lecturer at the Department of Chemical Engineering at Imperial College London. He is co‐Founder of the fabriCELL centre for artificial cell research and co‐Director of the Membrane Biophysics Platform. Yuval received his PhD in 2015 from the Department of Chemistry at Imperial, which was followed by several independent fellowships. His undergraduate training was at Cambridge, where he studied natural sciences. His research interests are in the development of artificial cell tools and technologies for clinical and industrial applications*.